# The Impact of Supratrochlear Foramen on Humeri’s Medullary Canal Diameter and Its Surgical Implications

**DOI:** 10.7759/cureus.43864

**Published:** 2023-08-21

**Authors:** Prabhjot K Chhabra, Swati Yadav, Rajesh K Jangir, Ritubhi Mehta, Mahindra Anand

**Affiliations:** 1 Anatomy, Mahatma Gandhi Medical College and Hospital, Jaipur, IND; 2 Anatomy, Santosh Medical College and Santosh Deemed to be University, Ghaziabad, IND; 3 Radiography, Mahatma Gandhi Medical College and Hospital, Jaipur, IND

**Keywords:** oval supratrochlear foramen, supratrochlear foramen, translucency of septum, anatomical variations of humerus, medullary canal of humerus

## Abstract

Introduction

Supracondylar elbow fractures are prevalent in the pediatric age group, and retrograde intramedullary nailing of the humerus is a common treatment approach. The anatomy of the medullary canal and the presence of the supratrochlear foramen (STF) significantly influence the stabilization of the nail. This study aimed to determine the incidence and morphology of the STF and compare the width of the medullary canal in humeri with and without the STF.

Methods

We examined 40 humeri bones with fused humeral epiphyses for the presence of the STF obtained from the Department of Anatomy's osteology collections. We studied the morphology and measured the dimensions of the foramen. We then compared the morphometric characteristics of humeri bearing the STF with those without it. We also took radiographs of all bones and measured the medullary canal width at three levels. We compared the measured width of the medullary canal in humeri between those with an STF and those without. This analysis was performed using IBM SPSS Statistics version 27.0 (Armonk, NY: IBM Corp.) for Windows and an unpaired t-test. We considered p-values of less than 0.05 as statistically significant.

Results

We found the STF in six bones (15%) as follows: four on the left side and two on the right. Humeri with the STF had smaller mean head circumference, length, and shaft circumference than those without the foramen. However, this difference was not statistically significant (p>0.05). The mean width of the medullary canal in humeri with the STF was significantly smaller (p<0.05) than in bones without the STF.

Conclusions

Our study revealed that humeri with an STF present a significantly smaller diameter of the medullary canal, which can impact surgical procedures like retrograde intramedullary nailing. Clinicians should consider this when planning surgeries to avoid iatrogenic fractures and enhance procedural efficiency. Our data also suggest that larger humeri are less likely to have an STF, potentially influencing pre-surgical planning and fracture risk assessment.

## Introduction

Supracondylar fractures of the elbow are frequently observed in the pediatric population [1]. Midshaft and distal humeral fractures usually occur secondary to trauma in adults. The common treatment for these fractures is retrograde intramedullary nailing of the humerus. However, the distal part of the humerus, which has a narrow medullary cavity, is considered a critical zone for potential iatrogenic fractures during this procedure [2]. Iatrogenic comminution, fissures, or avulsions can occur during retrograde intramedullary nailing [3]. The consideration of the humerus’s medullary canal anatomy and the presence of a supratrochlear foramen (STF) are crucial to improving nail stabilization [4]. Bones that contain an STF typically have a short and narrow medullary canal [5].

The presence of an STF (also referred to as the inter-condylar foramen or septal aperture) varies among global populations. The foramen is observed in approximately 7-11% of the Turkish population [6,7], 11% of Koreans [8], 15.8% of Europeans [5], 10.5% of Chinese [9], 34.4% of Indians [10], and 47% of Africans [11]. Several shapes have been recognized, including oval, round, triangular, sieve-like, reniform, and irregular. This study aimed to examine the incidence and morphology of the STF, compare the morphometric characteristics of the humerus with and without a septal aperture, and assess the width of the medullary canal in bones with and without an STF.

## Materials and methods

**Figure 1:**
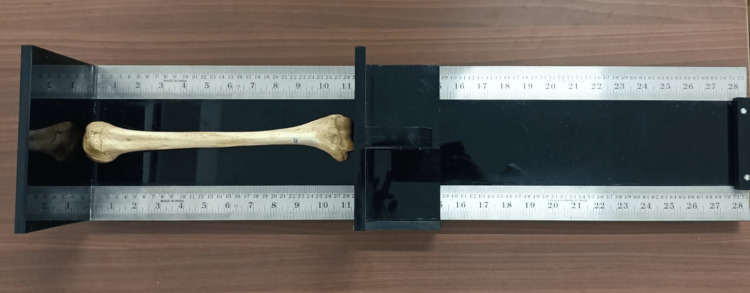
Measurement of length of humerus with osteometric board.

This study was conducted in the Departments of Anatomy and Radiology at Mahatma Gandhi Medical College and Hospital, Jaipur, following approval from the Institutional Ethics Committee (#MGMC&H/IEC/JPR2022/691). We studied 40 humerus bones with fused humeral epiphyses obtained from the Department of Anatomy's osteology collections. These bones were sourced from cadavers donated for research and study. Bones showing signs of injury, bony lesions, neoplasia, or damage were excluded. Each bone was labeled from one to 40, and the side of the bone and the presence of the STF were noted. Intercondylar width and bone length were measured. The length of the bone was measured as the distance between the highest point on the head of the humerus to the lowest point on the trochlea. An osteometric board (made up of black acrylic 10 mm, measuring bones up to 28×6.75 inches) was used to measure the length of the bone (Figure 1).

**Figure 2:**
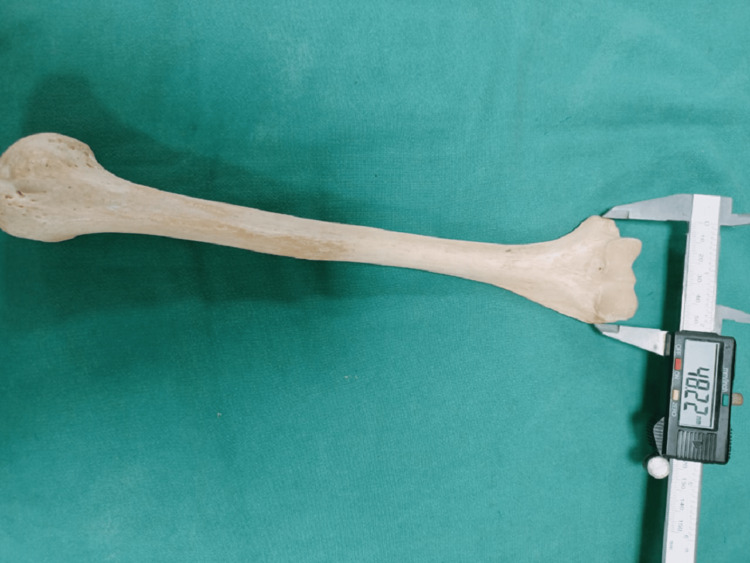
Measurement of intercondylar width of humerus.

**Figure 3:**
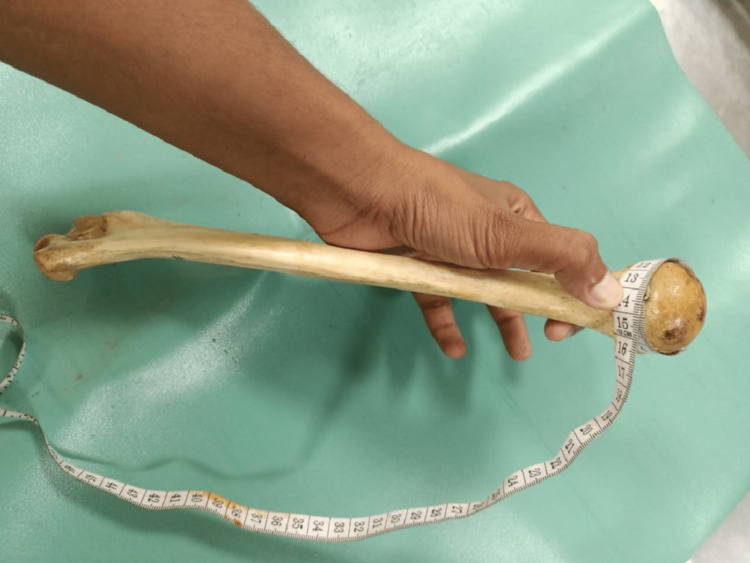
Measurement of head circumference of humerus.

Intercondylar width was measured as the distance between the medial and lateral epicondyle of the humerus using digital vernier calipers (Figure 2). The circumferences of the head and shaft were determined using a measuring tape. Shaft circumference was measured at three following positions: the proximal 25th percentile, midshaft 50th percentile, and distal 75th percentile marks of the humeral length [12]. Head circumference was measured as the perimeter along the outer edge of the head of humerus at its junction with anatomical neck (Figure 3).

For bones containing an STF, the shape was categorized as oval, round, sieve-like, reniform, or irregular. We used a digital vernier caliper (150 mm/6 inch, stainless steel model) to measure the maximum transverse and vertical diameters of oval and reniform shapes. The lower end of humerus has a septum between coronoid and olecranon fossa. This septum is either opaque, translucent, or a foramen is visible. In bones lacking a foramen, we assessed the translucency of the septum between the coronoid and the olecranon fossa by positioning the humerus’s lower end against an x-ray lobby/view box.

Radiographs of all bones were taken in an anteroposterior view to measure the medullary canal. Software-assisted measurements of the intramedullary canal width were conducted at three following levels: below the surgical neck of the humerus, the middle of the shaft below the deltoid tuberosity, and the lower end of the medullary cavity of the shaft using Masterview DICOM 4.5.3 Beta software (Pune, India: Medsynaptic Pvt Ltd).

Statistical analysis

We used an unpaired t-test to compare the mean dimensions of the humerus bones with and without an STF. The mean width of the medullary canal, as measured in radiographs, was compared between bones with and without an STF using an unpaired t-test, and we considered p<0.05 as statistically significant. Statistical analyses were conducted using IBM SPSS Statistics version 27.0 (Armonk, NY: IBM Corp.) for Windows.

## Results

**Figure 4:**
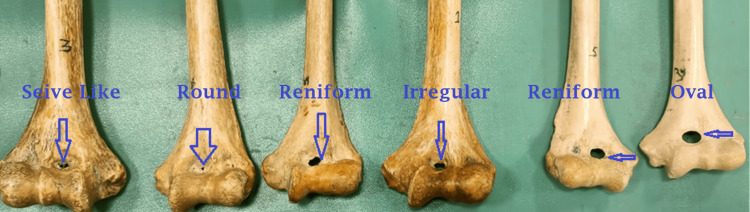
The lower end of the humerus shows different shapes of the supratrochlear foramen.

**Figure 5:**
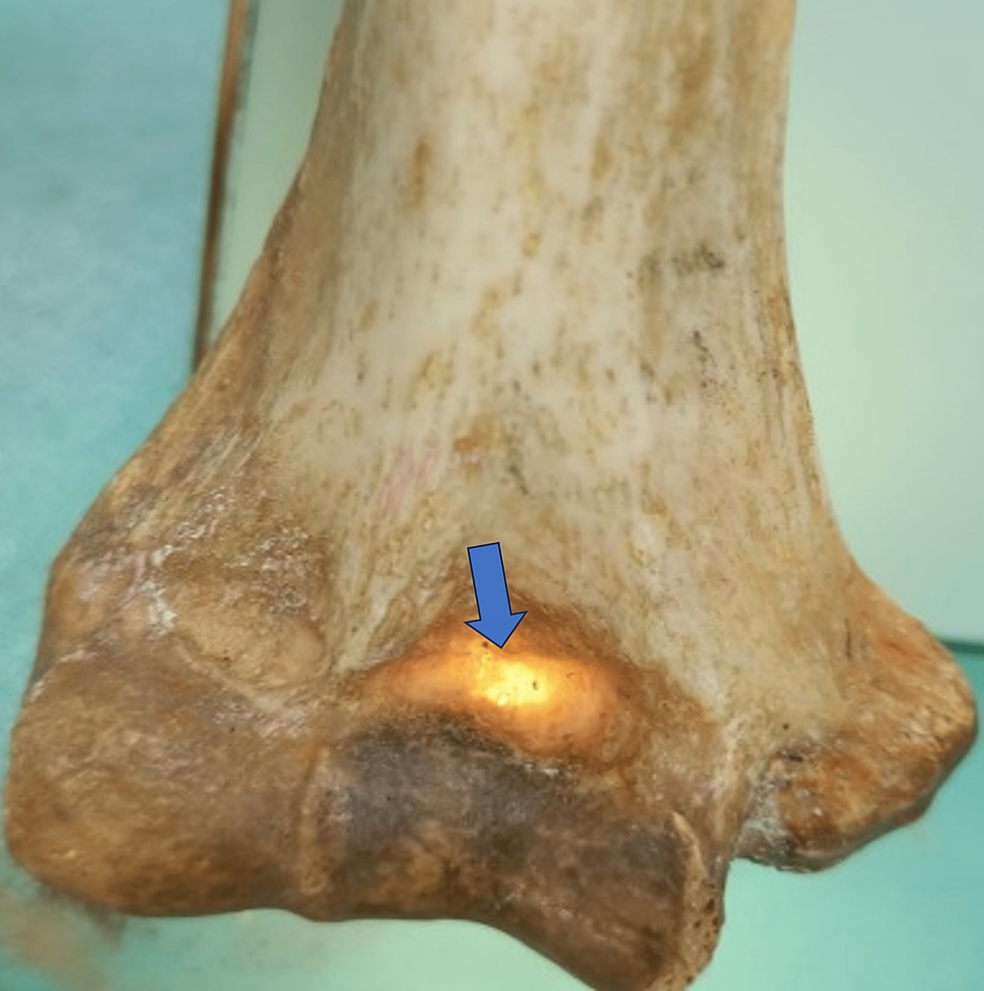
The lower end of the humerus shows translucency of the septum.

This study involved 40 dried humerus bones, 22 from the left side and 18 from the right. An STF was observed in six bones (15%), four from the left and two from the right (Table 1, Figure 4). In the remaining 34 specimens, the septum between the coronoid and olecranon fossa was translucent (Figure 5).

**Table 1:** Incidence of structural differences in supratrochlear foramen. STF: supratrochlear foramen

STF shape	STF Location	
	Right side, n (%)	Left side, n (%)
Sieve like	1, (2.5%)	0
Round	0	1, (2.5%)
Reniform	1, (2.5%)	1, (2.5%)
Irregular	0	1, (2.5%)
Oval	0	1, (2.5%)
Translucency of septum	18, (45%)	16, (40%)

**Figure 6:**
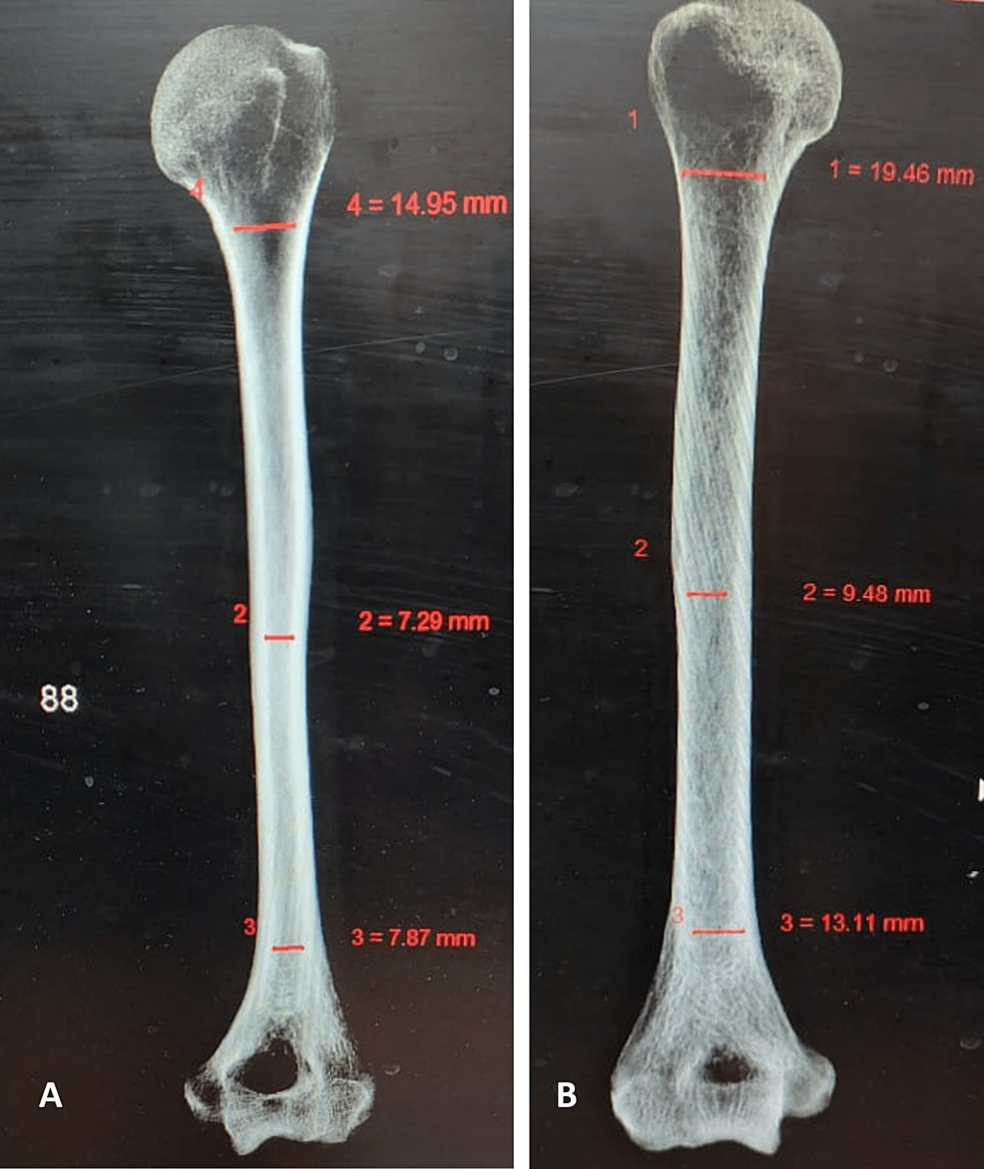
Radiographs of humeri bones show the width of medullary canal in bone with (A) and without (B) supratrochlear foramen.

The mean transverse diameter for oval and reniform shapes was 8.17 mm, and the average vertical diameter was 5.27 mm. Table 2 compares the dimensions of humeri bones with and without an STF. In Table 3, we present the results of our comparison of the mean width of the intramedullary canal in bones with and without STF, revealing a significantly smaller canal width in bones with the foramen. Figures 6A, 6B illustrates these differences with radiographs of humeri bones, displaying the reduced width of the medullary canal in the bone with an STF compared to the bone without the STF.

**Table 2:** Comparison of dimensions of humeri bones with and without STF. STF: supratrochlear foramen

Measurements	Characteristics of humerus		t-test	p-Value
	Without STF	With STF		
Mean humeral length	342±88.77 mm	297.90±14.56 mm	1.20	0.23
Mean shaft circumference, upper	20.89±4.54 mm	19.30±4.35 mm	0.795	0.431
Mean shaft circumference, middle	18.78±4.66 mm	18.77±4.55 mm	0.010	0.992
Mean shaft circumference, lower	19.20±4.57 mm	19.04±7.24 mm	0.072	0.943
Intercondylar width	59.61±7.72 mm	53.55±4.70 mm	1.851	0.072
Mean head circumference	149.50±38.76 mm	141.19±10.68 mm	0.517	0.608

**Table 3:** Comparison of mean width of intramedullary canal in bones with and without STF. *P-value<0.05 is considered statistically significant. STF: supratrochlear foramen

Measurement location	Mean width of intramedullary canal		t-test	p-Value
	With STF	Without STF		
Surgical neck of humerus	10.38±3.36 mm	16.99±5.25 mm	-2.961	0.005*
Middle of shaft (below deltoid tuberosity)	5.89±2.61 mm	7.84±1.90 mm	-2.193	0.034*
Lower end of medullary canal	6.88±1.19 mm	10.32±3.0 mm	-2.746	0.009*

## Discussion

Various etiological factors contribute to the formation of the STF. A positive correlation exists between its formation and a lack of humerus robusticity [13]. Myszka reported a higher frequency of this foramen in the right humerus of males [14] although other studies found it more common on the left side and predominantly in females [15].

Pathological or segmental humerus shaft fractures usually require open reduction and internal fixation. Highly displaced type-3 supracondylar fractures can be treated with intramedullary fixation of the humerus, which can be optimized with an anatomical evaluation of the medullary canal. The humerus’s endosteal canal is funnel-shaped, wider at the top, and narrower at the bottom. This narrowing continues until the isthmus, where the diameter remains constant until the supracondylar region. The isthmus appears at the junction of the shaft’s upper two-thirds and lower third [4]. As measured in anteroposterior radiographic views in their study, the narrowest segment ranged from 5 to 10 mm [4]. In this study, the mean diameter below the deltoid tuberosity was 5.89 mm in bones with an STF and 7.84 mm in those without.

The mean diameter of the medullary canal was significantly smaller in bones with an STF compared to those without, a difference found to be functionally significant (p<0.05). Veerappan reported a mean diameter of the intramedullary canal as 6.36 mm in bones with an STF and 4.48 mm in those without [16].

Pospula et al. found the mean diameter was 9.1±2.4 mm below the deltoid tuberosity and 9.9±2.8 mm at the lower end of the medullary canal [17]. This study also noted a slight increase in the mean diameter of the medullary canal from 5.89 mm to 6.32 mm at the lower end. Furthermore, a good view of the STF can be obtained with a radiograph setup of 45 Kv and 0.08 mA and by increasing the distance of the x-ray tube [18].

In this study, the predominant shapes observed were oval and reniform, with no triangular foramen noted. Bahşi found 22 foramina (11 on each side) in 108 humerus bones in Turkey, with oval being the most common shape [19]. STF appears as a radiolucent area in the radiogram of upper limb and can be confused with a cystic or osteolytic lesion [20]. Knowledge of different shapes of foramen might help to reduce errors in the interpretation of bony lesions at lower end of humerus by radiologists.

Humerus bones with an STF had a higher head circumference (149.50+38.76 mm) than those without (141.19±10.68 mm). Ndou and Schepartz found a significant difference in head circumference in humerus bones with an STF in Black patients [12]. The mean robusticity index (length/diameter) of bones with an STF (17.236) was higher than that of bones without (16.80). Despite our small sample size, the humerus bones with an STF had a higher mean humeral length, head circumference, epicondylar width, and shaft circumference than those without. Ndou and Schepartz suggested that genes for bone size and larger body frame might result in a phenotype that has less probability of STF formation [12].

Our study had several important limitations. First, the sample size was relatively small, which might affect the robustness of our findings and limit the generalizability of the results. The study was based on dried humerus bones, so the outcomes might not fully represent the characteristics of living humerus bones. Our study did not account for potential demographic variables, such as age, sex, or ethnic background, which could influence the formation of an STF. Additionally, we did not examine the potential biomechanical implications of the presence of the STF. Future research with larger, more diverse sample sizes and in vivo investigations are needed to substantiate and extend our findings.

## Conclusions

This study aimed to investigate the prevalence and anatomical features of STF in the humerus and to explore the impact of its presence on the medullary canal’s diameter. Our findings revealed that bones with an STF exhibit a significantly smaller medullary canal diameter, a potentially critical factor in procedures involving the humerus. These findings bear important implications for medical practice. When planning for retrograde intramedullary nailing, the presence of an STF should be considered to prevent iatrogenic fractures and improve the procedure’s efficiency. Furthermore, our results suggest that the larger the humerus bone the fewer the chances to have an STF, a potentially influential factor in pre-surgical planning and fracture risk assessment.
